# Metabolomics Analysis Reveals Interaction of Base-Line Chemotherapy and Shiyiwei Shenqi Tablets in Breast Cancer Treatment

**DOI:** 10.3389/fphar.2021.720886

**Published:** 2021-09-10

**Authors:** Hong Wan, Xiaojun Xu, Xiaowei Yang, Angqing Li, Xiaopeng Ma, Aman Xu, Xiao Yuan, Wenbin Wang, Tao Guo, Guangtao Luo, Xiaobo He, Wang Li, Zhaorui Wang, Qiang Sun, Jing Pei, Yongzhen Guo, Yong Zhu

**Affiliations:** ^1^ Department of General Surgery, The Fourth Affiliated Hospital of Anhui Medical University, Hefei, China; ^2^ Department of Breast Surgery, The First Affiliated Hospital of Anhui Medical University, Hefei, China; ^3^ Department of General Surgery, The First Affiliated Hospital of Anhui Medical University, Hefei, China; ^4^ Department of Thyroid and Breast Surgery, The First Affiliated Hospital of University of Science and Technology of China, Hefei, China; ^5^ Department of Head and Neck Surgery, The First Affiliated Hospital of University of Science and Technology of China, Hefei, China; ^6^ Cancer Institute, Xuzhou Medical University, Xuzhou, China; ^7^ Department of Pathology, The Third Affliated Hospital of Zhengzhou University, Zhengzhou, China

**Keywords:** shiyiwei shenqi tablets, breast cancer, untargeted metabolomics, amino acid metabolism, side effects

## Abstract

Shiyiwei Shenqi Tablet (SSTs) has been widely used for treatment of different types of cancer including breast cancer. SST has drawn more and more interest due to the low rate of side effects. The aim of this study was to investigate the metabolites in serums of breast cancer patients who received base-line chemotherapy only or combination treatment with SST. An untargeted metabolomics method was developed to investigate the alteration of metabolism in patients’ serums using ultra-high-performance liquid chromatography/Q-exactive Orbitrap mass spectrometry. The patients were separated based on the metabolomics data, and further analyses showed that SST treatment can affect the metabolism of glucose, fatty acid, bile acid and amino acid. In particular, SST treatment significantly reduced some short peptides which are potential tumor neoantigens. This study may provide novel insights into the mechanism underlying interaction between SST and base-line chemotherapy in terms of affecting metabolic pathways and thereby changing metabolic products, which might shed new light for clinical medication.

## Introduction

Breast cancer is the leading female cancer (2,261,419 cases worldwide in 2020) and the leading cause of cancer-related deaths around the world (684,996 deaths worldwide in 2020) (DeSantis et al., 2019; Sung et al., 2021). Despite dramatic improvements achieved in breast cancer diagnosis and treatments, the prognosis of breast cancer patients, especially those with metastasis, remains unsatisfactory (Miller et al., 2019). In China, the breast cancer incidence and mortality are rising (Chen et al., 2016). According to the report of Centers for disease Control of China, breast cancer has become the most common cancer type in Chinese women. There were 416,371 new breast cancer cases in 2020 in China, and 117,174 breast cancer-induced death in 2020, which impose a heavy burden on the public health system (Cao et al., 2021). The influence of some recognized risk factors such as alcohol use, tobacco use, hormone levels, and body weight on breast cancer generation, and the roles of metabolism in breast cancer etiology and treatment remain unclear and need further study (Li et al., 2016).

Metabolomics is characterized as a technique that can comprehensively and simultaneously identify small molecule metabolites and quantify their changes under different conditions (Johnson et al., 2016; Cui et al., 2018; Jang et al., 2018; Guo et al., 2019). Metabolomics aims to identify indicators to reflect interactions between biological systems. So, it is an ideal holistic method for investigation of drug-drug interactions to obtain further insights into pharmacodynamic mechanisms (Kalwat and Cobb, 2017). Non-targeted metabolomics provides high-throughput analysis of metabolites in samples, which is very helpful for investigating new drug-drug interactions (Li et al., 2017; Zhang et al., 2020). Several analytical platforms including nuclear magnetic resonance (NMR), high performance liquid chromatography mass spectrometry (HPLC-MS), and gas chromatography mass spectrometry (GC-MS) are used for non-targeted metabolomics analysis (Alonso et al., 2015). MS based metabolomics approaches have been widely applied due to their high throughput and sensitivity, and highly quantitative and reproducible data (Cui et al., 2018). Furthermore, the negative and positive ionization also enhances the examination sensitivity.

Although surgery chemotherapy, radiotherapy, and targeted therapy have been combined and widely utilized for breast cancer treatment, recurrence and metastasis remain leading to a high mortality of advanced breast cancer patients (Harbeck et al., 2019). Furthermore, chemotherapy can lead to substantial side effects as well as drug resistance, resulting in therapeutic failure eventually (Ponde et al., 2019). Therefore, it is urgent to seek novel treatment methods for breast cancer. Traditional Chinese medicine (TCM) has been widely used to treat various types of cancers (Yan et al., 2017; Xiang et al., 2019), particularly, TCM has been applied in almost half patients who have breast cancer in China (Poo et al., 2020; Yang et al., 2021). Notably, the utilization of TCM in Western countries is also rising. Previous studies showed that the extract of *Astragalus* membranaceus can induce apoptosis of several breast cancer cells *via* suppressing PI3K/AKT/mTOR pathway (Zhou et al., 2018; Liu et al., 2019). Scutellaria barbata plus Hedyotis diffusa has shown high efficacy in breast cancer therapy (Fang et al., 2020). These studies indicate the promising prospects of TCM in breast cancer therapy.

TCM has drawn more and more interest due to its high safety and efficacy, low toxicity and side effects, as well as potential synergistic effects when combined with chemotherapy (Xiang et al., 2019). Shiyiwei Shenqi Tablet (SST) is a traditional Chinese decoction that has been utilized for treatment of leucopenia and relief of chemotherapy related symptoms: lassitude, vomiting, weakness, nausea, emaciation, and dizziness. SST consists of 11 Chinese herbs including Panax ginseng C. A. Mey, *Astragalus* abbreviatus Kar. and Kir, Angelica acutiloba (Siebold & Zucc.) Kitag, Gastrodia elata Blume, Rehmannia glutinosa (Gaertn.) DC, Catsia tora Linn, Rhizoma Alismatis, Cuscuta abyssinica A. Rich, Asarum acuminatum (Ashe), E. P. Bicknell, Cornu Cervi, and Lycium chinense Mill) (Dong et al., 2010; Chen et al., 2011; Yang et al., 2017). The mechanisms of pharmacodynamics and drug-drug interaction for SST still remain unclear. Moreover, the influence of compounds on metabolism of each other is very complicated. Therefore, new strategy is needed to investigate the drug-drug interactions in combination treatment with base-line chemotherapy and SST. In this study, we performed non-targeted metabolomics analysis using HPLC-MS to explore the interactions between base-line chemotherapy and SST in breast cancer treatment. Patients with sole base-line chemotherapy treatment or combination treatments were prospectively enrolled, and the changes of metabolites in serum were examined to elucidate the mechanism of interaction between base-line chemotherapy and SST.

## Materials and Methods

### Study Subjects

30 Chinese female patients aged from 30 to 70 years with a definite diagnosis of breast cancer were prospectively enrolled. The clinicopathological characteristics of the patients involved were assessed independently by two senior pathologists. In addition, patients with any prior treatment for breast cancer, preinvasive carcinoma, distant metastasis, other malignancies, active rheumatism, or heart failure were excluded. All the involved patients were informed about the participation benefits and risks, and were provided written informed consent. Studies were approved by the Institutional Review Board of the Fourth Affiliated Hospital of Anhui Medical University, and conducted in compliance with the Declaration of Helsinki principles (PJ-YX2020-011).

### Study Design

The 30 patients were randomly divided into two groups: 1) base-line chemotherapy treatment group (n = 15); 2) adjuvant SST treatment group (n = 15). Patients from the adjuvant SST treatment group were given epirubicin combined with cyclophosphamide as the neoadjuvant chemotherapy and SST. Patients from the base-line chemotherapy treatment group received epirubicin combined with cyclophosphamide as neoadjuvant therapy. The dosages of epirubicin and cyclophosphamide were 90 mg/m^2^ and 600 mg/m^2^ per course, respectively. The dosage of SST was 2 g three times a day. Before treatment and 14 days after treatment, the whole blood samples were clot and centrifuged for 10 min at 1,000×g, and serum was isolated and stored at −80 °C until further use.

### Formula of SST

First, Panax ginseng C.A.Mey [Araliaceae; Panax quinquefolius var. ginseng], 67 g *Astragalus* mongholicus Bunge [Fabaceae; Astragalus membranaceus Fisch. ex Bunge], 89 g Angelica sinensis (Oliv.) Diels [Apiaceae; Angelica polymorpha var. sinensis Oliv], 44.5 g Gastrodia elata Blume [Orchidaceae; Gastrodia elata var. gracilis Pamp], 89 g Rehmannia glutinosa (Gaertn.) DC. [Orobanchaceae; Rehmannia Libosch. ex Fisch. and C.A.Mey.], 66.5 g Alisma plantago-aquatica subsp. orientale (Sam.) Sam. [Alismataceae; Alisma plantago-aquatica var. orientale Sam.], 89 g *Senna* tora (L.) Roxb. [Fabaceae; *Cassia* contorta Vogel], 22 g Cornu Cervi Pantotrichum [Cervidae; antler of *Cervus* nippon Temminck], 66.5 g Cuscuta chinensis Lam. [Convolvulaceae; Pentake chinense (Lam.) Raf.], 2.5 g Asarum sieboldii Miq. [Aristolochiaceae; Asarum sieboldii var. cornutum Y.N.Lee], 66.5 g Lycium barbarum L. [Solanaceae; Lycium barbatum Thunb.]. All of these herbs were grounded into powder. The powder were then boiled at high pressure for 20 h. Further, the residues were filtrated, and the paste was concentrated to a relative density of 1.20–1.25 (55–60°C) at reduced pressure, then spray-dried and crushed into fine powder. Finally, the sucrose were added to the powder to make 1000 g SST.

### Untargeted Metabolomics Analysis

*Sample preparation:* The metabolites in serum were identified using HPLC-MS. For HPLC-MS analysis, 200 µL serum sample was added into a tube with 1,200 µL ice methanol and acetonitrile (50/50, v/v). After a 10-min standing at -20°C, the solution was centrifuged for 10 min at 10000 rpm. Then the supernatant was dried by rotary evaporation, and 50 µL acetonitrile was added for re-dissolving and further analysis by LC-MS.

*LC-MS conditions:* LC experiment was conducted on a waters e2695 UPLC (waters, the USA). A Fortis C18 column (2.1 × 100 mm, 1.7 μm) was used at 40°C. Water containing acetic acid (99.9/0.1, v/v, solvent A) and acetonitrile (solvent B) were used as mobile phase. A gradient of 0 min, 5% (B); 1 min, 25% (B); 3 min, 45% (B); 6 min, 95% (B); and 12 min, 95% (B) was used in both positive and negative mode. The posting time was set as 2 min. The injection volume and flow rate of the mobile phase were 5 μL and 0.3 ml/min, respectively. Electrospray ionization (ESI) with both negative and positive full scan was used for detection. Solutions were infused at 0.3 ml/min with the following parameters: temperature: 350°C, capillary: 4000 V, and speed of drying gas using nitrogen: 12 L/min. The samples for quality control (QC) were prepared by mixing all the serum samples from patients and injected six times before test for system stability checking and also injected every 10 samples during test.

### Data and Statistical Analysis

The untargeted metabolomics was carried out as described previously (Sha et al., 2020). Briefly, deconvolution of LC-MS spectrum, including peak alignment, noise processing and baseline correction was performed using Thermo Data Analysis software. Compound Discoverer TM2.0 was then used for normalization to obtain the matrix. The raw intensities were transformed and normalized. For multiple peaks mapped to the same metabolites, the average intensity values were used. The matrix was then subjected to orthogonal partial least squares discrimination analysis (OPLS-DA) and principal component analysis (PCA) using the R ropls package (version 1.21.0) to obtain the differential metabolites between groups (Thevenot et al., 2015). Metabolites with variable importance in projection (VIP) > 1 were further analyzed by one-way analysis of variance (One-way ANOVA), followed by Benjamini-Hochberg correction and fold change analysis. Metabolites with fold change >2 or <0.5 and FDR <0.05 were considered to have statistically significant difference. Finally, the metabolites were traced to metabolite pathways through the Kyoto encyclopedia of genes and genomes (KEGG) by MetaboAnalyst (https://www.metaboanalyst.ca/MetaboAnalyst/home.xhtml) (Chong et al., 2018). All the statistics were performed using the R software (version 3.4.4).

## Results

### Identification of Key Compounds in SST

Serum samples from 30 breast cancer patients were used for untargeted metabolomics analysis. Among them, sample 1 and 3 from base-line chemotherapy and adjuvant SST treatment groups failed in untargeted metabolomics analysis due to their relatively low data quality. Baseline characteristics are presented in Table 1. The clinical characteristics including age, tumor TNM stage, tumor size, and PR, ER, HER2 and lymph node metastasis status showed no significant differences between the two groups to ensure the accuracy of this study. We used magnetic resonance imaging (MRI) to evaluate the efficacy of the two groups, and the results showed the SST group exhibited a better efficacy on breast cancer after a 14-day treatment (Supplementary Figure S1).

**TABLE 1:** Demographic and baseline disease characteristics of the patients.

Characteristic	SST group (N = 12)	Base line group (N = 14)	*p* Value
**Age**			ns
<45 yr	5	6	-
≥45 yr	7	8	
**ER status**			ns
Positive	6	7	-
Negative	6	7	
**PR status**			ns
Positive	7	8	-
Negative	5	6	
**Her-2 status**			ns
Positive	4	5	-
Negative	8	9	
**Tumor size**			ns
≤2 cm	2	3	-
>2 cm	10	11	
**Nodal status**			ns
Positive	12	13	-
Negative	0	1	
**TNM stage**			ns
Stage II	8	9	-
Stage III	4	5	

Yr = *years*; p *value was calculated using Chi-square* test.

**FIGURE 1:**
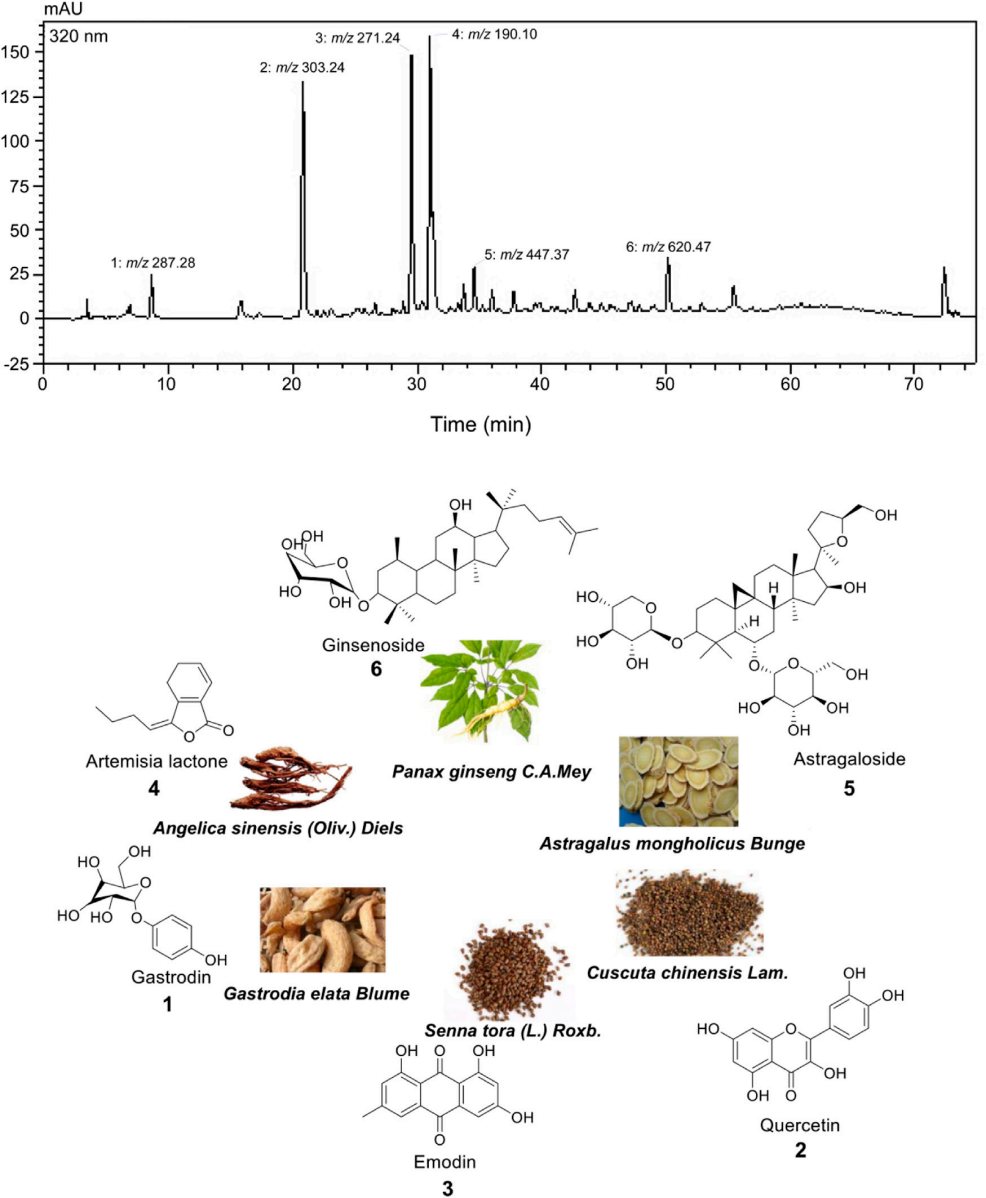
Structures of compounds identified from Shiyiwei Shenqi Tablets.

SST is mainly composed of eleven traditional Chinese medicine ingredients. Through our detection, we have found six key components including ginsenoside, artemisia iactone, astragaloside, gastrodin, emodin, and quercetin in SQEI by LC-HRMS (Figure 1). Therefore, we considered these six compounds as the main pharmaceutical components.

### Untargeted Metabolomics Analysis of Serum Samples After Base Line Treatment

To confirm the stability of our HPLC-MS system, we performed PCA on the untargeted metabolomics data based on both positive and negative ion models. The results showed that the QC samples could be discriminated from the real samples in both positive and negative ion models. Furthermore, the QC samples tended to be clustered, which demonstrated the stability of our HPLC-MS system (Supplementary Figure S2A). We also calculated the relative standard deviation (RSD) of each metabolite identified. The results showed that the RSD values of different peaks of QC samples were small, further confirming the stability of our HPLC-MS system (Supplementary Figure S2B).

**FIGURE 2:**
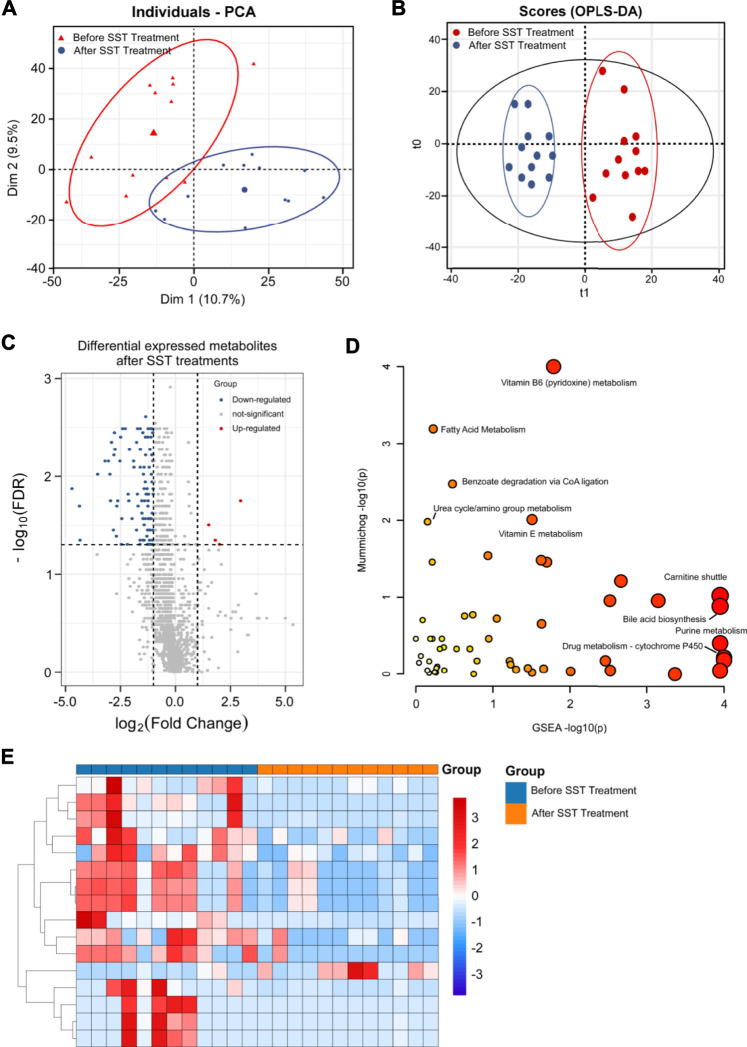
Metabolomics analysis of breast cancer patients’ serums after base-line chemotherapy. **(A, B)** PCA **(A)** and OPLS-DA **(B)** plots of the metabolites detected in serums of breast cancer patients before and after base-line chemotherapy (n = 14). **(C)** Volcano plot showing the DEMs identified in serums of breast cancer patients after base-line chemotherapy. **(D)** The bubble plot showing the enriched pathways of DEMs identified in serums of breast cancer patients after base-line chemotherapy. **(E)** The heatmap illustrating the levels of DEMs identified in serums of breast cancer patients after base-line chemotherapy; each row represents one metabolite, and each column represents one sample.

We also performed untargeted metabolomics analysis using an HPLC-QE-Orbitrap-MS platform for 14 serum samples from breast cancer patients who received base line chemotherapy before surgery. Serum samples from patients before and after base line chemotherapy treatment were subjected to metabolomics analysis using an optimized HPLC-QE-Orbitrap-MS method. The raw MS data of both negative and positive modes were imported into Thermo Data Analysis software for peak detection, correspondence, normalization and alignment. A data matrix was then subjected OPLS-DA and PCA using R ropls package. The PCA score plot (Figure 2A) showed that serum samples after base line chemotherapy could be discriminated from those before base line chemotherapy (R2X = 0.526). Further OPLS-DA confirmed that serum samples after base line chemotherapy were clearly distinguished from those before base line chemotherapy (Figure 2B), and the Q2Y and R2Y values were 0.883 and 0.984, respectively.

VIP, which is frequently used in metabolomic analysis, was employed to identify the differentially expressed metabolites (DEMs) after treatments. High-resolution MS peaks were annotated to known metabolites by Thermo Data Analysis software according to the database including HMDB, KEGG, and in-house reference metabolites. The VIP score and fold change for each metabolite before and after treatments were then calculated. In total, 186 metabolites including 41 up-regulated and 145 down-regulated metabolites showed significant changes after treatments (Figure 2C and 2E, FDR <0.05 and fold change >2 or <0.5 and VIP >1) (the complete DEMs list is shown in Supplementary Table S1). The DEMs included amino acid, fatty acids and phospholipids, such as 1-[(9Z)-hexadecenoyl]-sn-glycero-3 -phosphocholine, [FA (18:4)]6Z_9Z_12Z_15Z-octadecatetraenoic acid, Ala-Pro, and Gly-Pro (Table 2).

**TABLE 2:** The representative differential metabolites identified in serum samples after base-line treatment.

Compound	m/z	*R*_*t*_	Molecular formula	VIP	FC	FDR	Change	Metabolic pathway
**1-(4Z,7Z,10Z,13Z,16Z,19Z-docosahexaenoyl)-sn-glycero-3-phosphocholine**	567.3312	5.744	C_30_H_50_N O_7_P	2.023	0.252	0.0014	Down	Phospholipid metabolism
**1-[(11Z,14Z)]-icosadienoyl-sn-glycero-3-phosphocholine**	547.3628	6.401	C_28_H_54_N O_7_P	2.006	0.426	0.0022	Down	Phospholipid metabolism
**1-[(8Z,11Z,14Z)-icosatrienoyl]-sn-glycero-3-phosphocholine**	545.3476	6.131	C_28_H_52_N O_7_P	1.110	0.470	0.0468	Down	Phospholipid metabolism
**1-[(9Z)-hexadecenoyl]-sn-glycero-3-phosphocholine**	493.3160	5.68	C_24_H_48_N O_7_P	1.596	0.430	0.0261	Down	Phospholipid metabolism
**Ala-Pro**	186.1001	0.688	C_8_H_14_N_2_ O_3_	1.232	4.006	0.0152	Up	Amino acid metabolism
**Gly-Pro**	172.0848	0.79	C_7_H_12_N_2_ O_3_	1.375	2.656	0.0152	Up	Amino acid metabolism
**H-Gly-Pro-Pro-OH**	269.1370	0.877	C_12_H_19_N_3_ O_4_	1.208	3.268	0.0261	Up	Amino acid metabolism
**leu-leu-leu-leu**	470.3460	2.624	C_24_H_46_N_4_ O_5_	1.792	0.276	0.0019	Down	Amino acid metabolism
**Linoleamide**	279.2556	6.829	C_18_H_33_NO	1.339	0.382	0.0082	Down	Fatty acid metabolism
**Methyl L-leucyl-** **l** **-leucinate**	258.1939	2.429	C_13_H_26_N_2_ O_3_	1.785	0.217	0.0026	Down	Amino acid metabolism
**N5-Ethyl-** **l** **-glutamine**	174.1004	1.706	C_7_H_14_N_2_ O_3_	1.547	2.301	0.0082	Up	Amino acid metabolism
**N-Acetyl-D-alloisoleucine**	173.1044	2.816	C_8_H_15_NO_3_	1.759	2.403	0.0026	Up	Amino acid metabolism
**N-Acetyl-DL-norvaline**	159.0886	2.227	C_7_H_13_NO_3_	1.834	2.234	0.0011	Up	Amino acid metabolism
**PC**	757.5612	13.71	C_42_H_80_N O_8_P	1.987	0.406	0.0064	Down	Phospholipid metabolism
**Val-Arg**	273.1799	2.594	C_11_H_23_N5 O_3_	1.041	2.604	0.0173	Up	Amino acid metabolism
**[FAtrihydroxy**(**18:0)]9_10_13-trihydroxy-11-octadecenoicacid**	330.2402	4.596	C_18_H_34_O_5_	1.639	0.356	0.0046	Down	Fatty acid metabolism
**[FA**(**18:4)]6Z_9Z_12Z_15Z-octadecatetraenoicacid**	276.2083	5.782	C1_8_H_28_O_2_	1.813	0.433	0.0040	Down	Fatty acid metabolism
**[FAhydroxy**(**20:4)]5S-hydroxy-12-keto-6Z_8E_10E_14Z-eicosatetraenoicacid**	334.2132	7.307	C_20_H_30_O_4_	1.942	0.172	0.0011	Down	Fatty acid metabolism
**(Dichloromethyl)phosphonate**	161.9056	0.656	CHCl_2_O_3_P	1.642	0.203	0.0298	Down	Phospholipid metabolism
**Val-Leu-Gly-Lys**	415.2773	2.575	C_19_H_37_N5 O_5_	1.171	2.502	0.0229	Up	Amino acid metabolism

The changes of metabolic pathways were analyzed using MetaboAnalyst webtool, and the results showed that the potential metabolic pathways related to base line chemotherapy were those involved in metabolism of amino acids, fatty acids, and vitamins. In particular, pathways involved in metabolism of glycosphingolipid, linoleate, and leukotriene, and carnitine shuttle were considered the most probably involved metabolic pathways after base line chemotherapy (Figure 2D and Supplementary Table S2).

### Untargeted Metabolomics Analysis of Patient Serums After SST Treatment

**FIGURE 3:**
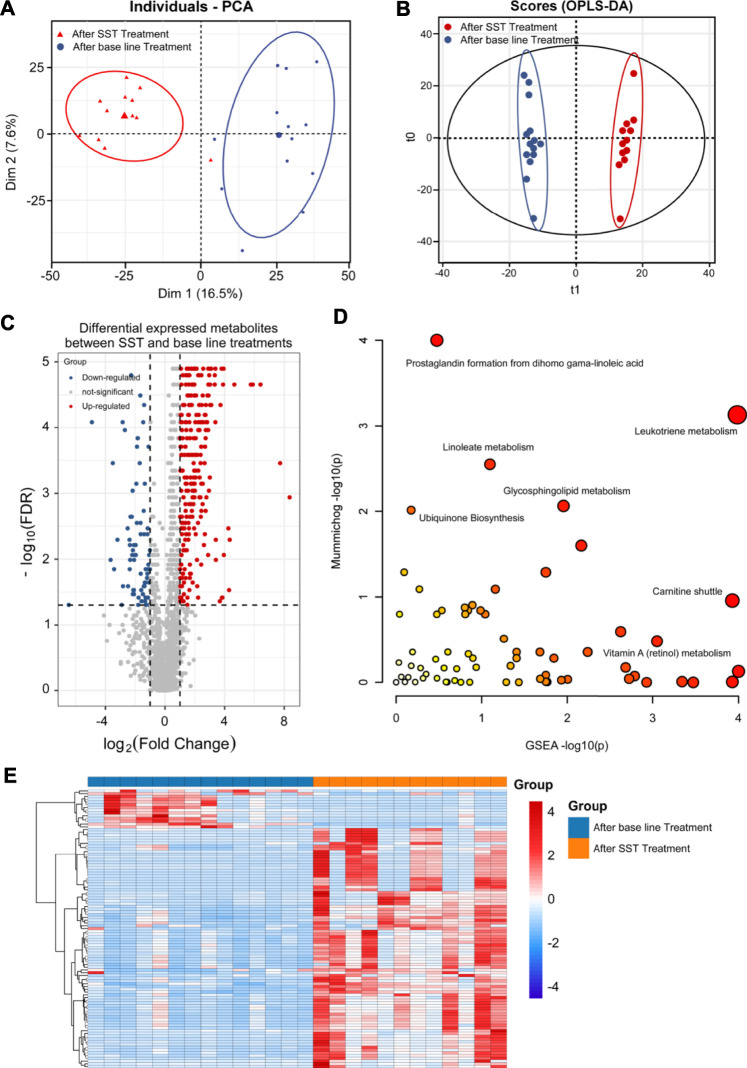
Metabolomics analysis of breast cancer patients’ serums after SST treatment. **(A, B)** PCA **(A)** and OPLS-DA **(B)** plots of the metabolites detected in serums of breast cancer patients before and after SST treatment (n = 12). **(C)** Volcano plot showing the DEMs identified in serums of breast cancer patients after SST treatment. **(D)** The bubble plot showing the enriched pathways of DEMs identified in serums of breast cancer patients after SST treatment. **(E)** The heatmap illustrating the levels of DEMs identified in serums of breast cancer patients after SST treatment; each row represents one metabolite, and each column represents one sample.

Untargeted metabolomics analysis of patient serum samples was conducted using HPLC-QE-Orbitrap-MS platform. After peak detection, alignment, correspondence, and normalization, the PCA score plot showed that the serum samples after SST treatment could be distinguished from those before SST treatment (Figure 3A, R^2^X = 0.540). Further OPLS-DA confirmed that serum samples after SST treatment were clearly discriminated from those before SST treatment, and the R^2^Y and Q^2^Y values were 0.921 and 0.583, respectively (Figure 3B).

To identify the DEMs after SST treatment, the high-resolution MS peaks of the LC-MS chromatograms were annotated to known candidates by Thermo Data Analysis software according to the database including HMDB, KEGG, and in-house reference metabolites. The VIP score for each metabolite was then calculated by combing the peaks mapped to one metabolite. Totally 76 down-regulated and 3 up-regulated metabolites exhibited significant changes after SST treatment (Figure 3C and 3E, FDR <0.05 and fold change >2 or <0.5 and VIP >1) (the complete DEMs list is shown in Supplementary Table S3). Interestingly, two dipeptides His-Trp and L-Phe-L-Phe were significantly up-regulated in serum after SST treatment (Table 2). In contrast, several metabolites involved in metabolism of nucleotides such as adenine and adenosine were dramatically down-regulated after SST treatment (Table 3).

**TABLE 3:** The representative differential metabolites identified in serum samples after SST treatment.

Compound	m/z	*R*_*t*_	Molecular formula	VIP	FC	FDR	Change	Metabolic pathway
**His-Trp**	341.1479	1.815	C_17_H_19_N_5_O_3_	1.594	7.792	0.018	Up	Amino acid metabolism
**Cystathionine**	222.0672	2.883	C_7_H_14_N_2_O_4_S	2.110	0.109	0.007	Down	Amino acid metabolism
**Adenosine**	267.0962	0.872	C_10_H_13_N_5_O_4_	1.695	0.141	0.035	Down	Nucleotide metabolism
**Histidinate**	154.0620	2.086	C_6_H_8_N_3_O_2_	1.411	0.205	0.003	Down	Amino acid metabolism
**Arg-Phr**	321.1790	1.025	C_15_H_23_N_5_O_3_	1.560	0.427	0.015	Down	Amino acid metabolism
**Deoxycholic Acid**	392.2926	6.832	C_24_H_40_O_4_	1.818	0.496	0.015	Down	Bile acid metabolism
**L-Phe-L-Phe**	312.1470	3.003	C_18_H_20_N_2_O_3_	1.581	4.004	0.049	Up	Amino acid metabolism
**D-Ala-D-Ala**	160.0847	1.956	C_6_H_12_N_2_O_3_	1.502	0.495	0.013	Down	Amino acid metabolism
**Adenine**	135.0546	2.029	C_5_H_5_N_5_	2.142	0.488	0.004	Down	Nucleotide metabolism
**DL-α-Aminocaprylic acid**	159.1259	2.720	C_8_H_17_NO_2_	1.365	0.396	0.015	Down	Amino acid metabolism

We further analyzed the alterations of metabolic pathways after SST treatment using the MetaboAnalyst web tool. As shown in Figure 3D and Supplementary Table S4, the changes of metabolic pathways were mainly involved in metabolism of Vitamin B6 (pyridoxine), Vitamin E, and purine, carnitine shuttle, biosynthesis of bile acid, and regulation of drug metabolism-cytochrome P450. The scatter plot displayed the most possible metabolic pathways *via* circle size. Therefore, the metabolism of Vitamin B6 and purine, and carnitine shuttle were considered to have most altered metabolic pathways after SST treatment.

### Untargeted Metabolomics Analysis of Patient Serum Between SST Treatment and Base Line Chemotherapy

**FIGURE 4:**
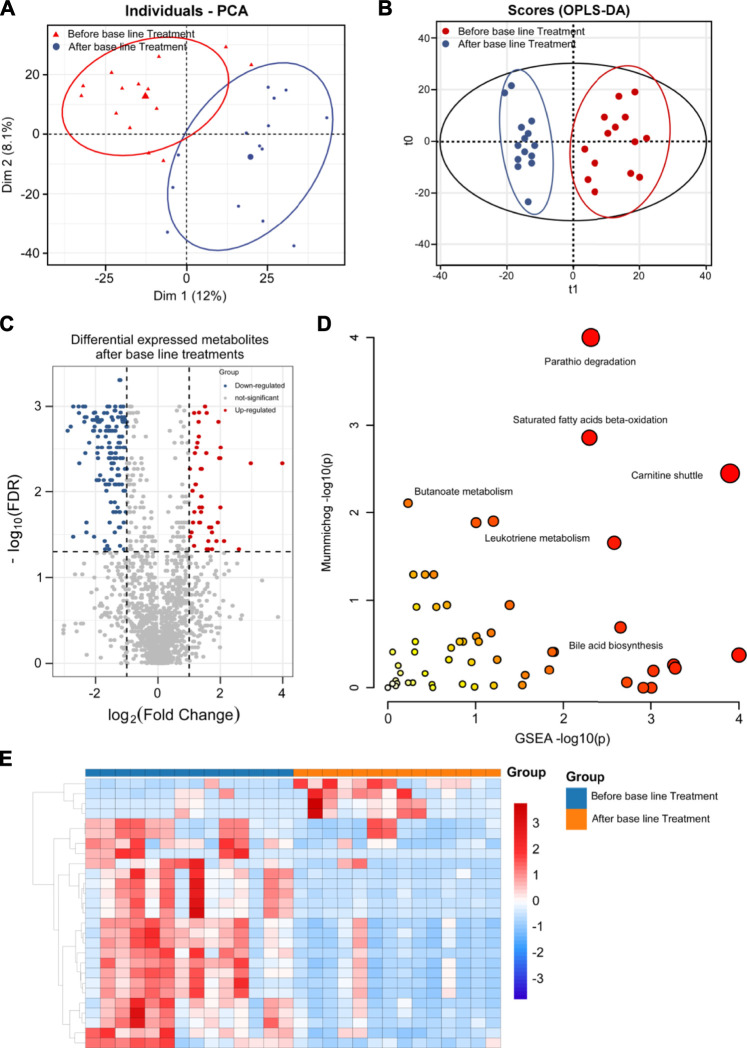
Metabolomics analysis of breast cancer patients’ serums between base-line chemotherapy and SST treatments. **(A, B)** PCA **(A)** and OPLS-DA **(B)** plots of the metabolites detected in serums of breast cancer patients between base-line chemotherapy and SST treatments (n = 12 for SST group, n = 14 for base-line chemotherapy group). **(C)** Volcano plot showing the DEMs identified in serums of breast cancer patients between base-line chemotherapy and SST treatments. **(D)** The bubble plot showing the enriched pathways of DEMs identified in serums of breast cancer patients between base-line chemotherapy and SST treatments. **(E)** The heatmap illustrating the levels of DEMs identified in serums of breast cancer patients between base-line chemotherapy and SST treatments; each row represents one metabolite, and each column represents one sample.

Since dramatic metabolic changes happened after SST treatment and base line chemotherapy, we next analyzed and compared these metabolic alterations between patients who received SST treatment or only base line chemotherapy. The PCA score plot clearly separated patients receiving SST treatment from those receiving base line chemotherapy based on the serum metabolomics data (Figure 4A, R^2^X = 0.522). The OPLS-DA results further demonstrated that serum samples after SST treatment were clearly discriminated from those after base line chemotherapy, the R^2^Y and Q^2^Y values were 0.898 and 0.655, respectively (Figure 4B).

DEMs were also identified by the VIP score of each metabolite. A total of 287 metabolites including 50 down-regulated and 237 up-regulated metabolites exhibited significant changes in serum between SST and base line chemotherapy treatments (Figure 4C and 4E, FDR <0.05 and fold change >2 or <0.5 and VIP >1) (the complete DEMs list is shown in Supplementary Table S5). In particular, metabolites such as phospholipids, amino acids, and fatty acids showed significant changes between SST and base line chemotherapy treatments (Table 4). The metabolic pathway changes between SST and base line chemotherapy treatments were further analyzed using the MetaboAnalyst web tool. As shown in Figure 4D and Supplementary Table S6, metabolic pathways involved in carnitine shuttle, saturated fatty acids beta-oxidation, parathio degradation, bile acid biosynthesis, and leukotriene metabolism were considered the most altered metabolic pathways between SST and base line chemotherapy treatments.

**TABLE 4:** The representative differential metabolites identified in serum samples between SST treatment and base-line treatment.

Compound	m/z	*R*_*t*_	Molecular formula	VIP	FC	FDR	Change	Metabolic pathway
**leu-leu-leu-leu**	470.346	2.624	C_24_H_46_N_5_	1.912	7.464	0.0001	Up	Amino acid metabolism
**LysoPC(20:5(5Z,8Z,11Z,14Z,17Z))**	541.3163	5.445	C_28_H_48_N_7_P	1.559	5.797	0.0007	Up	Phospholipid metabolism
**LysoPC(22:4(7Z,10Z,13Z,16Z))**	571.3629	6.335	C_30_H_54_N_7_P	1.670	2.926	0.0009	Up	Phospholipid metabolism
**LysoPC(22:5(7Z,10Z,13Z,16Z,19Z))**	569.347	6.013	C_30_H_52_N_7_P	1.651	3.764	0.0009	Up	Phospholipid metabolism
**N5-Ethyl-** **l** **-glutamine**	174.1004	1.706	C_7_H_14_N_23_	1.440	0.496	0.0073	Down	Amino acid metabolism
**N-Acetyl-** **l** **-methionine**	191.0612	2.264	C_7_H_13_NOS	1.285	0.447	0.0034	Down	Amino acid metabolism
**N-Acetyl-** **l** **-tyrosine**	223.0841	2.173	C_11_H_13_N_4_	1.496	0.464	0.0001	Down	Amino acid metabolism
**Omega**	321.2144	5.143	C_15_H_31_N_6_	1.180	3.107	8.23E-	Up	Fatty acid metabolism
**PC**	757.5612	13.71	C_42_H_80_N_8_P	1.671	2.589	0.0014	Up	Phospholipid metabolism
**Pyrimidine**	80.03746	11.15	C_4_H_4_N_2_	1.665	1.420	0.0018	Up	Nucleotide metabolism
**Stearic acid**	284.2714	8.208	C_18_H_36_O_2_	1.569	1.800	0.0050	Up	Fatty acid metabolism
**Ubiquinone-1(CoQ1)**	250.1202	5.035	C_14_H_18_O_4_	1.589	1.450	0.0014	Up	Energy metabolism
**val-leu-gly-lys**	415.2774	2.575	C_19_H_37_N_5_	1.045	0.271	0.0001	Down	Amino acid metabolism
**[FA**(**16:2)]N-hexadecyl-ethanolamine**	299.2819	5.945	C_18_ H_37_NO_2_	1.393	2.191	0.0004	Up	Fatty acid metabolism
**[FA**(**18:2)]9Z_11E-octadecadienoicacid**	280.2399	6.174	C_18_H_32_O_2_	1.713	1.846	0.0005	Up	Fatty acid metabolism
**[FA**(**18:4)]6Z_9Z_12Z_15Z-octadecatetraenoicacid**	276.2084	5.782	C_18_H_28_O_2_	1.669	3.871	0.0001	Up	Fatty acid metabolism
**Ala-Pro**	186.1001	0.688	C_8_ H_14_N_2_O_3_	1.292	0.154	0.0001	Down	Amino acid metabolism
**Arachidonic acid**	304.2401	7.147	C_20_H_32_O_2_	1.294	1.293	0.0086	Up	Fatty acid metabolism
**asp-gly-lys**	318.153	0.587	C_12_H_22_N_6_	1.500	6.844	0.0011	Up	Amino acid metabolism
**Creatine**	131.0697	0.706	C_4_H_9_N_3_O_2_	1.234	1.458	0.0034	Up	Amino acid metabolism
**Creatinine**	113.0591	2.904	C_4_H_7_N_3_O	1.581	1.860	0.0073	Up	Amino acid metabolism
**Gly-Pro**	172.0849	0.79	C_7_H_12_N_23_	1.173	0.415	0.0495	Down	Amino acid metabolism
**Lauryl glucoside**	348.2507	4.264	C_18_H_36_O_6_	1.237	1.522	0.0258	Up	Glycometabolism

### Amino Acid Metabolism Was Identified as an Important Pathway After SST Treatment by Untargeted Metabolomics Analysis

**FIGURE 5:**
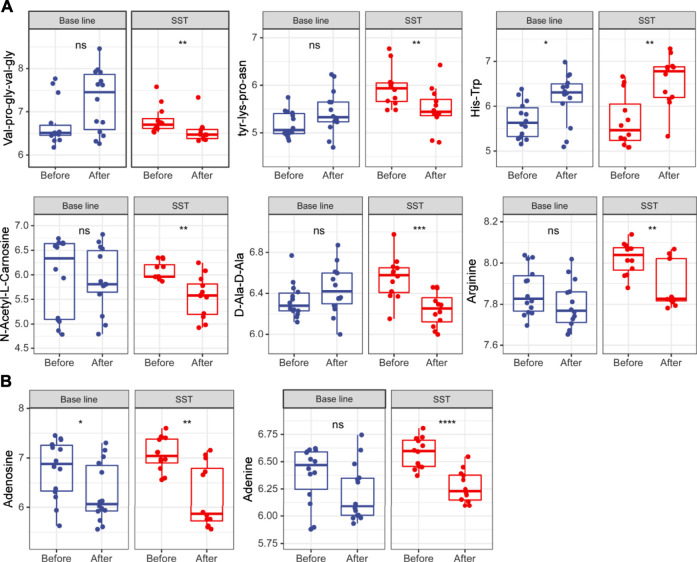
Levels of amino acid **(A)** and nucleotide **(B)** metabolism related metabolites in serums of breast cancer patients receiving base-line chemotherapy or adjuvant SST treatment; ns, not significant; *p < 0.05, **p < 0.01, ***p < 0.001, and ****p < 0.0001 by student’s *t* test.

Amino acid metabolism enhances tumor cell proliferation survival by supporting building block synthesis, producing immunosuppressive metabolites for immune escape, and reducing the production of agents mitigating oxidative stress (Pavlova and Thompson, 2016; Tabe et al., 2019). At the same time, numerous short peptides are generated during cancer initiation and progression. Untargeted metabolomics analysis results revealed that many short peptides such as val-pro-gly-val-gly, tyr-lys-pro-asn, and D-Ala-D-Ala were dramatically reduced after SST treatment, while these metabolites remained unchanged after base line chemotherapy (Figure 5A). In addition, some amino acids including N-Acetyl-l-Carnosine and arginine were also significantly reduced after SST treatment (Figure 5A). Intriguingly, the dipeptide his-trp was significantly increased after SST treatment, indicating SST treatment might induce the expression of some protective peptides (Figure 5A). Furthermore, some nucleotides such as adenine and adenosine were dramatically decreased after SST treatment compared with those after base line chemotherapy. These results showed that SST may exert its effect in breast cancer treatment through modulating amino acid metabolism.

### Glycometabolism Pathways Were Demonstrated as Co-regulatory Pathways in Both SST and Base Line Chemotherapy Treatments by Untargeted Metabolomics Analysis

**FIGURE 6:**
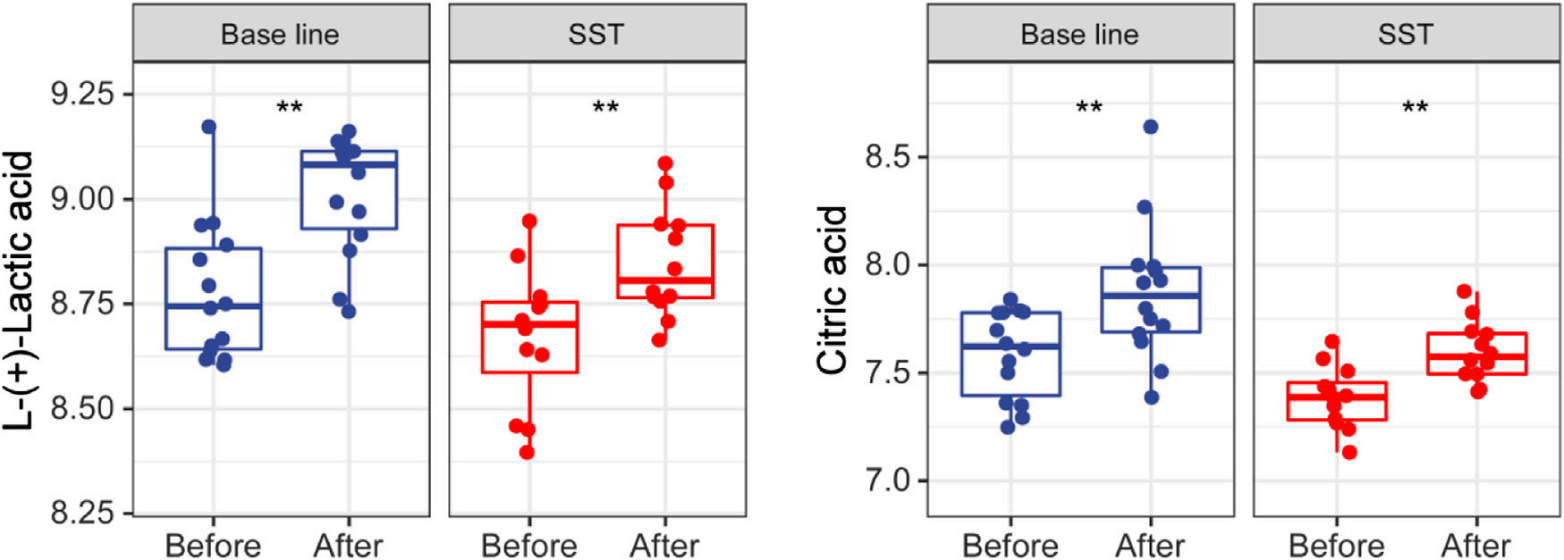
Levels of glucose metabolism related metabolites in serums of breast cancer patients receiving base-line chemotherapy or adjuvant SST treatment. **p < 0.01 by student’s *t* test.

Glycometabolism pathways including the tricarboxylic acid (TCA) cycle and glycolysis are reported to play important roles including promoting proliferation, growth, and long-term maintenance in carcinogenesis. (Pavlova and Thompson, 2016; Martinez-Outschoorn et al., 2017). The common characteristics of cancer cells known as Warburg Effect include enhancing uptake of glucose and glucose fermentation to lactate. Normal cells mainly produce energy through glycolysis, followed by TCA cycle and oxidative phosphorylation (Vander Heiden et al., 2009). However, cancer cells principally produce their energy through enhanced glycolysis, followed by lactic acid fermentation in the presence of abundant oxygen in microenvironment. Interestingly, citric acid and L-(+)-lactic acid were dramatically upregulated after both SST and base line chemotherapy treatments (Figure 6). Together, these upregulations may have resulted from cancer cells that are killed by chemotherapy or SST.

## Discussion

Nowadays, surgery combined with radiotherapy, chemotherapy, targeted therapy, hormonal therapy, and immunotherapy has been extensively used for treating patients with breast cancer (Harbeck et al., 2019). Chemotherapy is commonly used for treatment of different stages of breast cancer. Moreover, neoadjuvant chemotherapy has been used to shrink tumors before surgery (Early Breast Cancer Trialists' Collaborative Group (EBCTCG), 2018; Untch et al., 2014). Normally, the chemotherapeutic strategy is established by combining two or more agents to achieve a better effect for breast cancer. Although chemotherapy shows significant efficacy in killing cancer cells, it also results in non-negligible side effects such as alopecia, vomiting, constipation, diarrhea, immune dysfunction, and myelosuppression (Tao et al., 2015). The severity of these adverse effects is highly related to the regimen type, drug dosage, treatment length, and general health of patients. Furthermore, some breast cancer patients also show significant chemotherapy resistance. Thus, an adjuvant treatment aimed at managing side effects and increasing chemotherapy sensitivity is expected.

TCM has been increasingly combined with regular chemotherapy in breast cancer treatment because it can enhance chemotherapy sensitivity and alleviate the side effects induced by chemotherapy (Li et al., 2020). Some meta-analyses and systematic reviews have shown that the adjuvant use of TCM with chemotherapy can attenuate a series of side effects induced by chemotherapy including alopecia, vomiting, diarrhea, constipation, myelosuppression, and immune dysfunction (Li et al., 2020). For example, previous studies have shown that a TCM named Yiqi Jianpi Hewei could alleviate the occurrence rate of chemotherapy induced constipation in breast cancer patients (Wu et al., 2019; Li et al., 2020). The SST has been used to restore the immune function of chemotherapeutic or post-operational period gastric cancer patients (Lu et al., 2020). However, the mechanisms of pharmacodynamics and drug-drug interaction remain unclear. Moreover, the impacts of compounds on drug metabolism is not just simply the sum of effects from individual compounds.

In this study, we applied an untargeted metabolomics analysis method using HPLC-MS to examine the metabolite changes in serum of breast cancer patients following base line chemotherapy and SST adjuvant therapy. In addition, the mass spectrometry analysis data were further subjected to OPLS-DA and PCA analyses to obtain good division. Numerous differentially expressed metabolites especially those involved in fatty acid or amino acid metabolism have been identified after base line chemotherapy and SST adjuvant therapy, and treatments had shown significant impacts on the metabolites. We also analyzed the metabolic pathways involved based on the differentially expressed metabolites. The carnitine shuttle, bile acid biosynthesis, drug metabolism-cytochrome P450, and vitamin B6 (pyridoxine), Vitamin E, and purine metabolism pathways were considered as the potential pathways affected by SST treatment.

Amino acid metabolic disorder plays acritical role in breast cancer (Pavlova and Thompson, 2016; Tabe et al., 2019). The differentially expressed metabolites we found, such as val-pro-gly-val-gly, tyr-lys-pro-asn, and D-Ala-D-Ala were significantly reduced after adjuvant SST treatment. In breast cancer initiation and progression, many peptides called neoantigens are generated by disrupted alternative splicing processes and gene mutations (Benvenuto et al., 2019). In addition, amino acid metabolism disruption also leads to aberrant expression of short peptides in cancer. In this study, a lot of short peptides were detected with a remarkable decrease in breast cancer patients after adjuvant SST treatment. This result means that adjuvant SST treatment can inhibit the production of potential neoantigens, thereby alleviating tumor burden by affecting the amino acid metabolism disorder. Warburg effect has been known as an important phenomenon in cancer development: different from normal cells which primarily use oxidative phosphorylation in mitochondria to generate energy, most cancer cells primarily use aerobic glycolysis to obtain energy for development. We found the differential metabolites were all involved in aerobic glycolysis, such as L-(+)-lactic acid and citric acid, suggesting that the adjuvant SST treatment can improve the prognosis of breast cancer, thereby affect the glucose metabolism disorder.

We systematically performed untargeted metabolic analysis to investigate the potential mechanism of SST in breast cancer treatment. The results revealed that SST may enhance chemotherapy sensitivity and alleviate side-effects mainly by affecting the amino acid and glucose metabolism pathways. However, the detailed mechanisms of how SST impacts the cancer cell malignant biological properties remain further study. Furthermore, SST led to significant alleviation of side effects induced by chemotherapy, which may be related to drug and vitamin metabolism pathways. Hypothetically, SST may protect patients from vomiting and aspirating gastric contents through affecting the patients’ gut microbiota, thereby influencing the secondary metabolites.

At present, although TCM has been widely applied in breast cancer therapy for many years in China and also some other Asian counties (Xiang et al., 2019), it remains a challenge to introduce TCM to western countries. Currently, most reported clinical trials about the use of TCM in treating breast cancer were performed in Chinese patients. Therefore, more clinical trials are needed to be performed in western countries to facilitate the use of TCM worldwide. Another concern is whether TCM influences the pharmacokinetics of chemotherapeutic agents in combination treatments. Some *in vivo* studies showed that there were no interactions between chemotherapeutic agents and TCM such as berberine. However, pharmacokinetic studies of chemotherapeutic agents in combination with TCM are quite few. Moreover, the mechanisms about how TCM alleviates the side-effects induced by chemotherapy also need further research.

## Conclusion

We used an HPLC-MS-based untargeted metabolomics analysis method to investigate drug interactions between base line chemotherapy and SST treatment. Based on the HPLC-MS results, we have identified several differential metabolites after base line chemotherapy and SST treatment, respectively. The metabolites exhibited different changes before and after treatment of only base line chemotherapy or combination treatment with SST, indicating that the SST treatment can affect the glycometabolism, fatty acid, bile acid and amino acid metabolism. Especially, some short peptides which are potential tumor neoantigens were significantly reduced after adjuvant SST treatment. This work has elucidated the interaction mechanism between base line chemotherapy and SST treatment based on analysis of metabolite changes, and identified potential metabolic pathways involved, which might shed new light on clinical medication.

## Data Availability

The original contributions presented in the study are included in the article/Supplementary Material, further inquiries can be directed to the corresponding authors.
