# Supplementary material for: Metabolomics Analysis Reveals Interaction of Base-Line Chemotherapy and Shiyiwei Shenqi Tablets in Breast Cancer Treatment
Source: Front Pharmacol. 2021 Sep 10;12:720886. doi: 10.3389/fphar.2021.720886 (PMC8461015; doi:10.3389/fphar.2021.720886)
Supplement: Supplementary file 1 [file DataSheet2.docx]

**Supplemental Figure legends**


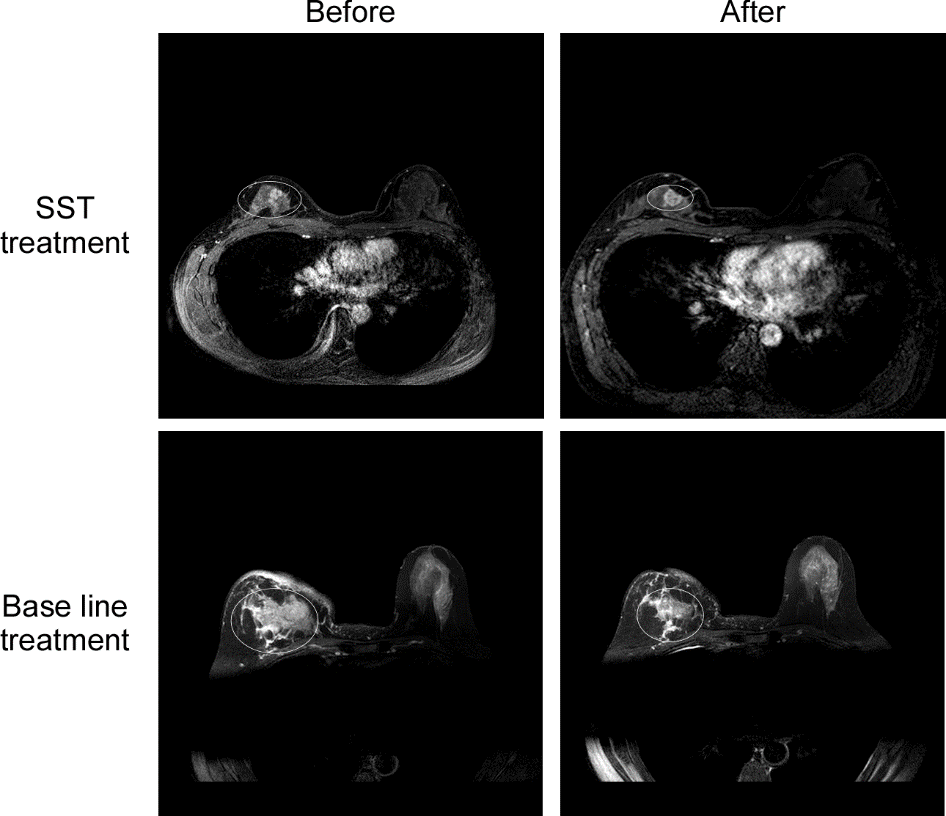


**Figure S1.** Representative MRI images of breast cancer patients after combined SST or base-line treatments.


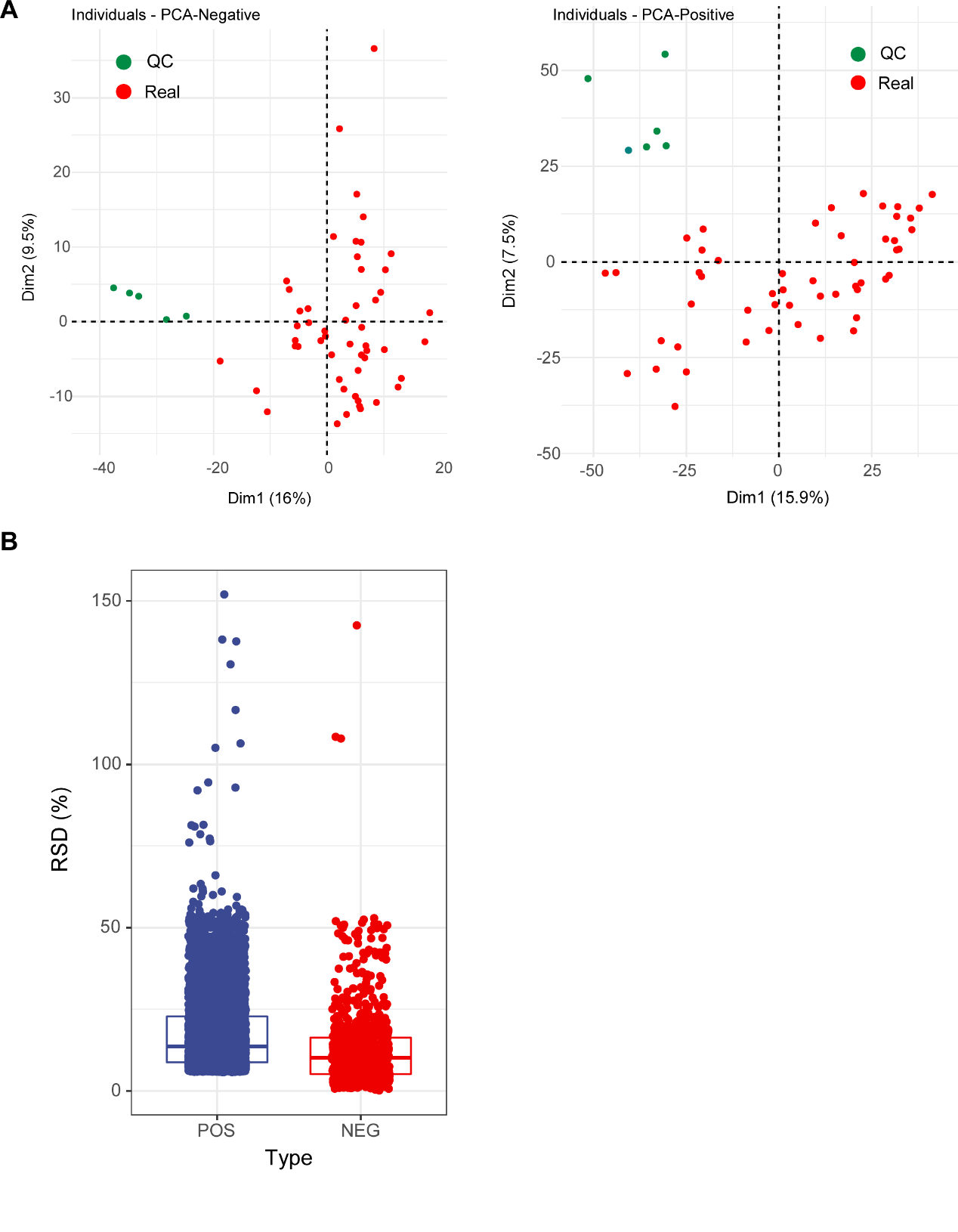


**Figure S2.** Quality control of the metabolomics data. **(A)** PCA plots of the QC and real samples in negative (left) and positive (right) ion models. **(B)** Boxplot of RSD value of identified metabolites in QC samples.
